# Design, synthesis, and characterization of novel Xc- transport inhibitors: Inhibition of microglial glutamate release and neurotoxicity

**DOI:** 10.21203/rs.3.rs-2932128/v1

**Published:** 2023-05-18

**Authors:** Mariusz Gajewski, Steven Barger

**Affiliations:** Arkansas Tech University; University of Arkansas for Medical Sciences

**Keywords:** Amino Acid Transport System Xc Protein, Cell Death, Drug Discovery, Glutamic Acid, Microglia, Stress, Oxidative

## Abstract

Neuroinflammation appears to involve some degree of excitotoxicity promulgated by microglia, which release glutamate via the system Xc- cystine-glutamate antiporter. With the aim of mitigating this source of neuronal stress and toxicity, we have developed a panel of inhibitors of the Xc- antiporter. The compounds were based on L-tyrosine, as elements of its structure align with those of glutamate, a primary physiological substrate of the Xc- antiporter. In addition to 3,5-dibromotyrosine, ten compounds were synthesized via amidation of that parent molecule with a selection of acyl halides. These agents were tested for the ability to inhibit release of glutamate from microglia activated with lipopolysaccharide (LPS), an activity exhibited by eight of the compounds. Two of these were further tested for the ability to inhibit death of primary cortical neurons in the presence of activated microglia. While both showed some neuroprotective activity, they were quantitatively distinct with a compound we refer to as “35DBTA7” showing the greatest effi cacy. This agent may hold promise in reducing the neurodegenerative effects of neuroinflammation in conditions such as encephalitis, traumatic brain injury, stroke, or neurodegenerative diseases.

## Introduction

Neuroinflammation contributes to many neurological conditions, including microbial infections, stroke, traumatic brain injury, age-related neurodegenerative diseases, and affective-state depression [1–4]. It is broadly defined as an inflammatory response within the brain or spinal cord; it usually arises from diseases, injuries, infections, or stress. Despite naturally positive outcomes of this state (e.g., tissue repair and recovery), there is a vast number of detrimental effects, some of which are devastating to the central nervous system (CNS). Regarding the example of stroke alone, approximately $34 billion a year is expended in healthcare services, medicines, and missed days of work [5]; the human cost of the associated long-term disabilities is immeasurable.

One of the remarkable mechanisms producing permanent CNS tissue damage is an acute or chronic release of high levels of an endogenous neurotransmitter—glutamate (Glu)—from microglia upon their activation [6]. Glu is the most prevalent excitatory neurotransmitter, crucial to all neurological circuits, and its receptors are abundant on many neurons. Excessive accumulation of Glu in extracellular fluid can cause toxic hyperexcitation of the cells: excitotoxicity. Excitotoxicity can cause CNS tissue damage, frequently progressive in nature, and is a serious long-lasting debilitating complication associated with neuroinflammation. While several products of activated microglia have been implicated in the neurodegenerative effects of neuroinflammation, we have determined that the vast majority of the neurotoxicity exhibited by activated microglia *in vitro* is dependent on their release of Glu via the system Xc- transporter [7–9].

System Xc- is an obligate-exchange transport protein, which expels Glu and imports cystine (Cys)_2_ through the plasma membrane [10]. This protein complex is localized on many cell types inside and outside of the CNS, including microglia. When activated, microglia produce abundant superoxide, consuming the cell’s major antioxidant: glutathione. Production of glutathione requires cysteine (Cys), which creates a Cys sink, quenched by (Cys)_2_ import. Because Xc- is the major mediator of (Cys)_2_ import, microglia dump Glu into the extracellular space as (Cys)_2_ follows this concentration gradient. This has the potential to impact extracellular Glu receptors in particular, which have an especially important role in excitotoxicity [11]. Considerable evidence indicates that this conversion of oxidative stress into an excitotoxic stress makes a substantial neurological impact [6, 8, 12, 13].

We developed a number of Xc- inhibitors and demonstrated and tested their ability to reduce microglial Glu release. We also carried two of these compounds forward in tests against neurotoxicity. Results indicate that analogs based on the structures discussed here could ameliorate the neuronal damage associated with CNS inflammation.

## Methods

### Drug design:

*De novo* design of potential inhibitors of system Xc- utilized computational methods, namely SYBYL 8.0 and Spartan ‘16 packages. Computational pharmacokinetics studies were powered by SwissADME model and besides bioavailability, the absorption and blood-brain barrier penetration properties were analyzed and graphed applying the *Brain Or IntestinaL EstimateD permeation* (BOILED- Egg) framework.

### Synthesis:

**Figure 1:**
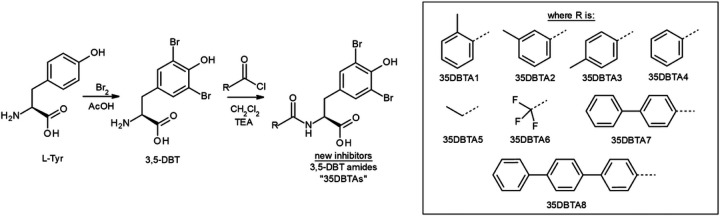
Design and scaffold of the first generation of prospective system Xc- inhibitors. L-Tyr reflects the shape of a locked conformer of L-Glu but has no activity at system Xc-. Halogenation of the aromatic ring of Tyr, followed by amidation of the molecule with a selection of acyl halides at 3,5-DBT’s α-amino group allowed several chemical structures to be tested, varying in a branch of the scaffold hypothesized to interact with a hydrophobic pocket in the enzyme.

Potential inhibitors of the Xc- transporter were synthesized by means of standard solution phase organic synthesis. Most of these were produced by electrophilic aromatic substitution/bromination of the aromatic ring of L-tyrosine (Tyr) to form 3,5-dibromotyrosine (3,5-DBT). This was followed by amidation of the α-amino group of 3,5-DBT with a selection of acyl halides. The latter was accomplished by adopting standard Schotten-Baumann approach. The synthesis leading to these molecules is outlined in Fig. 1. A 3,5-diiodo derivative of tyrosine (3,5-DIT) was also tested, as was 2,5-diiodohistidine (2,5-DIH).

### Characterization:

TLC of the products indicated the presence of contaminants. These were removed by dissolving the products in a diluted NaOH aqueous solution, followed by precipitation with 6 N HCl, twice. The precipitated products were dried, redissolved in ethyl acetate and resolved by chromatography on a preparatory silica plate in 5:5:1 ethyl acetate:hexane:methanol. The standard spectroscopic methods (MS, IR, ^1^H and ^13^C-NMR) were used to characterize the compounds before the focus was shifted to bioassays.

### Glutamate release assay:

Primary cultures of rat microglia were established as described previously [9]. Microglia were seeded at 375,000/cm^2^ in 96-well plates with minimal essential medium with Earle’s salts (MEM) containing 10% fetal bovine serum (FBS). After an overnight plating, the cultures were washed twice with serum-free MEM, and then a third volume was applied containing test substances at the indicated concentrations. Immediately thereafter, lipopolysaccharide (LPS) was added to some wells at 30 ng/ml. After 18 h, 50 μl of medium was transferred from each well to a black, opaque-bottomed 96-well plate and combined with 50 μl of assay reagent from a Amplex^®^ Red Glutamic Acid Assay kit (ThermoFisher). A standard curve generated with a range of concentrations of L-glutamate (monosodium salt). The assay reagent contained 0.25 U/ml horseradish peroxidase, 0.08 U/ml L-glutamate oxidase, 0.5 U/ml L-glutamate/pyruvate transaminase, and 200 μM L-alanine. After 30 min, fluorescence was measured in a Molecular Devices SpectraMax 3 with excitation at 550 nm and emission at 590 nm; values were interpolated into the standard curve. The cultures remaining in the original 96-well plates were combined with 3-(4,5-dimethylthiazol-2-yl)-2,5-diphenyl-2*H*-tetrazolium bromide (MTT) at a final concentration of 125 μg/ml and incubated for 30 min at 37°C and 5.5% CO_2_. After removal of the medium, 100 μl of dimethyl sulfoxide was added to dissolve the formazan crystals, and absorbance at 540 nm was measured in the SpectraMax 3.

### Neurotoxicity assay:

Primary cultures of rat cortical neurons were established as described previously [14], plated in 24-well plates. After the neurons had been cultured 8 days, primary microglia were plated in transwell inserts (12-mm diameter) at 375,000/cm^2^, washed to serum-free MEM, and treated with test substances followed immediately by LPS (100 ng/ml). After 1 h, the inserts were placed into the wells of the neuronal cultures. After an additional 18 h, the inserts were removed, and neuronal survival was evaluated by MTT assay as described above. Values are represented relative to the MTT signal produced in cultures exposed to untreated microglia.

## Results

The drug-design strategy was based on the structure of the fundamental, endogenous, transportable substrate Glu. Tyr was identified as a convenient scaffold molecule; 3D molecular modeling revealed a close similarity between Glu and Tyr, with the exception of the pKa of the distal carboxy group in Glu and the phenolic acidic proton in Tyr (~ 5 orders of magnitude difference in acidity). However, initial assays demonstrated that Tyr does not possess any inhibitory properties on the Xc- transporter, despite being a close mimic of Glu. Attention was then focused on increasing the acidity of the phenolic proton in Tyr, which led to the development of 3,5-dibromotyrosine (3,5-DBT) (Fig. 1). This compound was the first tyrosine-based Xc- inhibitor capable of potent inhibition of Xc- transport, and 3,5-DBT was used as a bioactivity reference molecule in subsequent assays of system Xc- activity. Even though the molecule is easy to synthesize and convenient in use (high activity, high solubility in water as a salt, and easily detected by MS due to presence of two bromine atoms), it is also very polar. Computational methods (SwissADME) and fundamental knowledge led to concerns that 3,5-DBT would be far too polar to cross the blood-brain barrier (BBB). Thus, efforts were focused on improving the pharmacodynamics through further modification of the amino acid. Previous structure-activity relationship (SAR) studies indicated that Xc- transporter has a large, lipophilic pocket that interacts with the α-amino side of 3,5-DBT. Prior experiments indicated that this amino group is likely dispensible for the compound’s inhibitory activity, so it may be considered an excellent attachment point to be exploited in subsequent lead optimization steps. This strategy proved very useful in manipulation of the pharmacokinetic properties of the new molecules until desired parameters were obtained. Several new 3,5-DBT analogs were prepared as 3,5-DBT α-amino amide derivatives (“35DBTA#”; Fig. 1).

**Figure 2:**
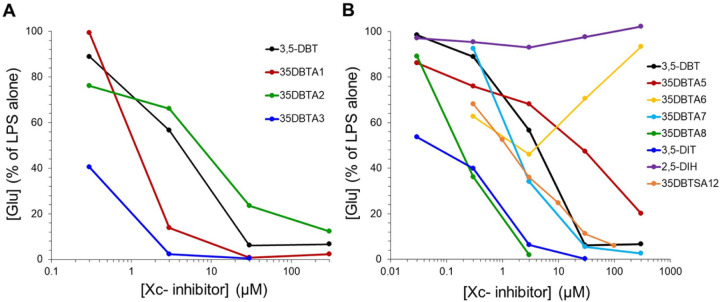
Microglial Glu release assay. Rat primary microglia were activated with 100 ng/ml LPS alone or immediately following application of the indicated concentrations of test substances. After 18 h in the presence of LPS, an aliquot of medium was assayed for Glu via a glutamate dehydrogenase colorimetric assay. A. The first generation of compounds was created by simply varying the position of a methyl group around an aromatic ring, comparing activity to 3,5-DBT. B. Additional modifications were evaluated, including several that simply extend the size of the structure hypothesized to fit into the hydophobic pocket of the Xc- transporter. Values are represented relative to the Glu detected in cultures treated with LPS alone; each data point represents a mean of 4 cultures.

The primary objective of developing Xc- transport inhibitors was to block the release of glutamate from activated microglia. This parameter was evaluated by assaying glutamate levels in the culture media of primary microglia exposed to LPS for 18 h (Fig. 2). Initial screening focused on structures wherein an aromatic ring was attached to the amide site, with a methyl group in various positions on the aromatic ring. Promising activity was observed with 35DBTA3, which has the methyl group in the *para*- position (Fig. 1). The *ortho-* and *meta-* analogs were effective, but 35DBTA3 displayed the highest activity (Fig. 2A).

**Figure 3:**
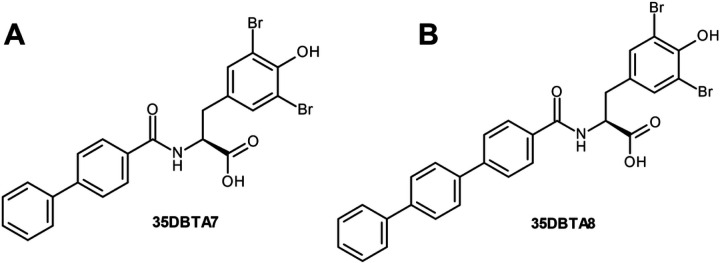
Structures of 35DBTA7 and 35DBTA8.

**Figure 4:**
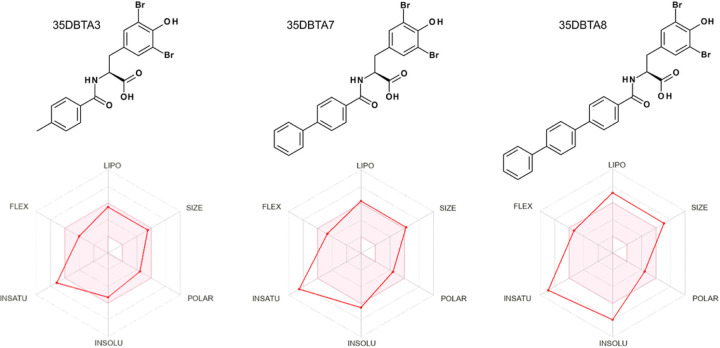
Drug-likeness and bioavailability of 35DBTA3, 35DBTA7 and 35DBTA8.

It remained possible that the methyl group at the *para-* position of 35DBTA3 was not necessarily providing the optimal interaction with the transporter’s lipophilic pocket. To further explore the architecture of the Xc- pharmacophore for optimizing the lead compound, another inhibitor was designed where an extension of the amide moiety by another aromatic ring was introduced. The simplest of these constructs was 35DBTA7 (Fig. 3A). Glu effl ux inhibition experiments demonstrated effi cacy for 35DBTA7 with an IC_50_ = 1.44 μM (Fig. 2B). Following same strategy, further extension of the amide side substituent with an additional aromatic ring produced 35DBTA8 (Fig. 3B). This new compound proved to be the most potent inhibitor in this series (IC_50_ = 183.5 nM concentration; Fig. 2B). However, the increased hydrophobicity of this molecule made it poorly soluble in aqueous solutions, rendering it impractical (Fig. 4).

Amides of 3,5-DBT are vulnerable to hydrolysis at the amide bond. Therefore, a new molecule was designed: 35DBTSA12, a sulfonamide analog of 35DBTA3, which was anticipated to be more stable. This compound exhibited an IC_50_ of 1.16 μM, similar to that of 35DBTA7 (Fig. 2B). To further explore the potential for amino acid derivatives to modulate system Xc-, we also tested 3,5-DIT and 2,5-DIH. While the latter was completely inactive, 3,5-DIT was approximately an order of magnitude more potent that 3,5- DBT (Fig. 2B).

**Figure 5:**
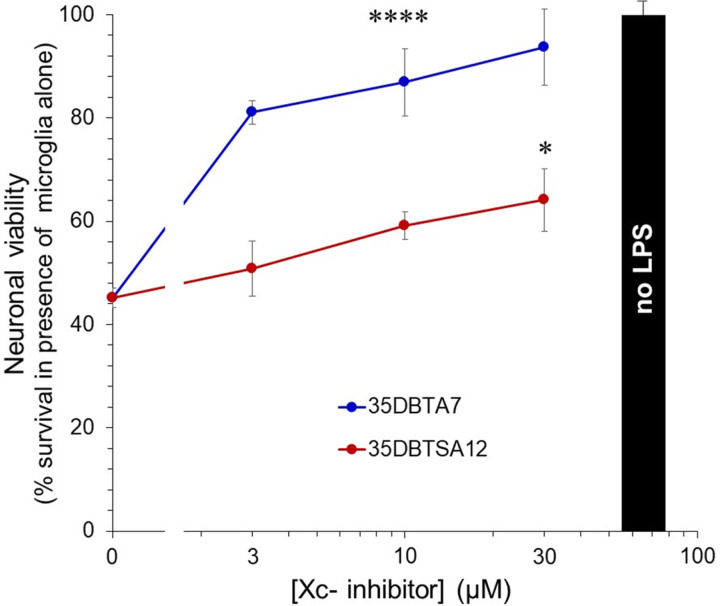
35DPTA7 and 35DPTSA12 reduce the neurotoxicity of activated microglia. Primary microglia were plated on permeable membranes in basket-type cell-culture inserts. Some cultures were treated with the indicated concentrations of 35DBTA7 or 35DBTSA12, followed immediately by application of LPS (100 ng/ml); some cultures were untreated and some received LPS alone. After 1 h, the inserts were placed into wells containing primary cortical neurons. After an additional 18 h, neuronal survival was determined by MTT assay. Values represent the percentage of the values obtained in wells containing untreated microglia (black bar); the y-intercept represents the neuronal viability values obtained in the presence of microglia treated with LPS alone. Values are mean ± SEM of quadruplicate cultures. ****P<0.0001 for all concentrations of 35DBTA7 versus LPS alone; *P=0.039 for 30 μM 35DBTSA12 versus LPS alone (ANOVA and Dunnett *post hoc*).

The ultimate objective of development of Xc- transport inhibitors for neuroinflammatory conditions is to reduce neurotoxicity under these conditions. To test the potential for such neuroprotection, 35DBTA7 and 35DBTSA12 were screened in a coculture model that allows agents secreted by microglia to diffuse to neurons without contact between the microglia and neurons. A considerable fraction of neurotoxicity found in microglial conditioned medium can be blocked with glutamate receptor antagonists [15, 16]. Primary microglia plated in transwell inserts were activated by LPS in the presence or absence of prospective Xc- inhibitors, then the microglial transwells were transferred to culture wells containing primary cortical neurons. Relative neuronal survival was determined 18 h later via MTT assay. Both 35DBTA7 and 35DBTSA12 elevated the survival signal (Fig. 5), likely through their documented suppression of glutamate release from the microglia. Despite the similar IC_50_ values for these two compounds in microglial release assays, 35DBTA7 was considerably more effective in neuroprotection than 35DBTSA12.

## Discussion

Microglia play important roles in neutralizing infectious pathogens, responding to traumatic or ischemic damage, and shaping synaptic/dendritic elements under conditions of neuroplasticity. However, excessive activation of microglia under conditions generally characterized as neuroinflammation can contribute to neurodegeneration [17]. Though neuroinflammation is a complex phenomenon associated with release of cytokines and other factors, a potent component of their neurotoxicity is the release of excitatory amino acid transmitters through system Xc- [6, 18–20]. Therefore, we have pursued the development of chemical agents that can inhibit Xc- transport, anticipating neuroprotection during conditions of

neuroinflammation. Approximately, a dozen compounds were designed, synthesized, and tested. LPS was used to activate microglia and induce their release of Glu. The analysis of 35DBTA7 and 35DBTSA12 was later extended to assays of neuroprotection in the face of activated microglia. Both compounds displayed their ability to reduce microglial release of excitotoxic levels of Glu *in vitro*.

Our early structure-activity relationship (SAR) studies implied that there is a large lipophilic pocket in the Xc- protein system on the side of the α-amino substituent of the inhibitors. After identification of the first inhibitor lead (3,5-DBT), a panel of new molecules for SAR studies was designed and prepared. Remarkable activity of 35DBTA3 (as compared to 35DBTA1, 35DBTA2, and 35DBTA4) drew the attention to the importance of the lipophilic substituent in the *para-* position of the aromatic system of the amide part. That position was later thoroughly explored. The new molecules, designated 35DBTA7 and 35DBTA8 were quite effective at inhibiting microglial glutamate release. It is important to point out that, besides the pharmacokinetics, one of the key factors guiding us in selection of promising inhibitors is their action at submicromolar concentrations. One inhibitor that did not follow trends was 35DBTA6, a trifluoroacetamide of 3,5-DBT. Above 3 μM this molecule lost inhibitory properties, which may be attributed to toxicity of fluorine derivatives, resulting in microglial lysis and indiscriminate leakage of Glu. Since one of the concerns was the possibility of premature hydrolysis of the new inhibitors (the amide bond), a stable sulfonamide 35DBTSA12 (a direct sulfo analog of 35DBTA3) was designed and tested. Even though its activity was similar to that of 35DBTA7, it showed diminished potency in the microglial glutamate release assay. 3,5-DIT displayed promising potency but proved to be exceedingly light sensitive and therefore less appealing.

To extend the relevance of the *in vitro* screening, the two compounds were tested in an assay of neuroprotection. Although other factors have been identified that mediate neurotoxicity in neuroinflammation, we find that Glu receptor agonists are among the most potent. For instance, neuroprotection in a microglia-neuron coculture was far superior with an inhibitor of nitric oxide synthase (NOS) 1—the neuronal isoform activated by Glu receptor agonism—than with an inhibitor of NOS2—the isoform expressed in activated microglia [7]. It is possible that Glu released via system Xc- is potentiated by other microglia derived Glu receptor ligands such as D-serine or quinolinic acid [21, 22]. Additionally, cytokines, reactive oxygen species, and proteases may play roles over distances in space and time [17]. Nevertheless, we and others find that a substantial fraction of microglial neurotoxicity can be alleviated by blocking the effects of Glu. Here, we found that 35DBTA7 was quite effective in such a model of indirect neurotoxicity. The lower effi cacy of 35DBTSA12 may indicate that it exerts a mild degree of direct neurotoxicity of its own. Alternatively, it is possible that 35DBTA7 actually served as a prodrug, hydrolyzing to 3,5-DBT for greatest effi cacy. If so, this relationship might be exploited *in vivo*, for instance, to delay the formation of 3,5-DBT until the prodrug has crossed the blood-brain barrier, thus trapping and concentrating the effective agent in the CNS.

**Figure 6:**
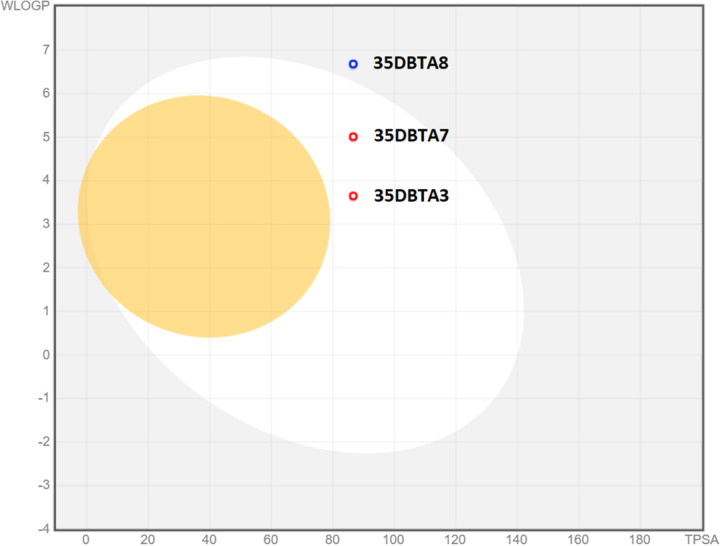
*Brain Or IntestinaL EstimateD permeation* method (BOILED-Egg) model portrays 35DBTA8 as a poor candidate for a drug that would partition to the CNS.

Because of their remarkable potency, 35DBTA3 and 35DBTA8 seem worth investigating further. However, 35DBTA8, being the best inhibitor from an activity standpoint, is computationally classified as having poor bioavailability, possibly not crossing the GI tract membrane (Fig. 4). Additionally, the *Brain Or IntestinaL EstimateD* (BOILED-Egg) permeation model portrays 35DBTA8 as a poor candidate for a drug that would partition to the CNS (Fig. 6). Relatedly, we encountered problems with solubility of this compound in aqueous media; even its disodium salt (carboxylate and phenoxide) was poorly soluble, producing turbid solutions/suspensions. This rendered 35DBTA8 impractical for our applications and narrowed the choices down to 35DBTA7 and 35DBTSA12. It may be worth noting that very potent inhibitors such as 35DBTA3 can have drawbacks, including effects that approach irreversibility and diffi culties in dosing across a diverse population.

The ultimate goal of these efforts is to treat human conditions having a neuroinflammatory component with system Xc- inhibitors—if not alone, then as part of a combinatorial therapy. Several clinical trials in humans which involved potential therapeutic agents have failed (especially in stroke), so this novel strategy seems worth investigating. The compounds identified here already serve as convenient molecular probes applicable in research on Xc- transport. Furthermore, the results of this study will guide the development of related optimized molecular tools useful for exploration of the Xc- pharmacophore. Neuroinflammation treatment *in vivo* studies utilizing a mouse model are in porogress.

## Data Availability

This study produced no data sets other than those presented in the figures.

## References

[1] Chen Z, Zhong D, Li G: The role of microglia in viral encephalitis: a review. J Neuroinflammation 2019, 16(1):76. 30967139 10.1186/s12974-019-1443-2 PMC6454758

[2] Kielian T: Immunopathogenesis of brain abscess. J Neuroinflammation 2004, 1(1):16. 15315708 10.1186/1742-2094-1-16 PMC516022

[3] Singh D: Astrocytic and microglial cells as the modulators of neuroinflammation in Alzheimer’s disease. J Neuroinflammation 2022, 19(1):206. 35978311 10.1186/s12974-022-02565-0 PMC9382837

[4] Wang H, He Y, Sun Z, Ren S, Liu M, Wang G, Yang J: Microglia in depression: an overview of microglia in the pathogenesis and treatment of depression. J Neuroinflammation 2022, 19(1):132. 35668399 10.1186/s12974-022-02492-0 PMC9168645

[5] Benjamin EJ, Blaha MJ, Chiuve SE, Cushman M, Das SR, Deo R, de Ferranti SD, Floyd J, Fornage M, Gillespie C : Heart Disease and Stroke Statistics-2017 Update: A Report From the American Heart Association. Circulation 2017, 135(10):e146-e603. 28122885 10.1161/CIR.0000000000000485 PMC5408160

[6] Barger SW: An unconventional hypothesis of oxidation in Alzheimer’s disease: intersections with excitotoxicity. Front Biosci 2004, 9:3286–3295. 15353358 10.2741/1481

[7] Barger SW, Basile AS: Activation of microglia by secreted amyloid precursor protein evokes release of glutamate by cystine exchange and attenuates synaptic function. J Neurochem 2001, 76(3 Part 3):846–854. 11158256 10.1046/j.1471-4159.2001.00075.x

[8] Barger SW, Goodwin ME, Porter MM, Beggs ML: Glutamate release from activated microglia requires the oxidative burst and lipid peroxidation. J Neurochem 2007, 101(5):1205–1213. 17403030 10.1111/j.1471-4159.2007.04487.x PMC1949347

[9] McMullan SM, Phanavanh B, Li GG, Barger SW: Metabotropic glutamate receptors inhibit microglial glutamate release. ASN Neuro 2012. 10.1042/AN20120044 PMC3413012 22770428

[10] Lewerenz J, Hewett SJ, Huang Y, Lambros M, Gout PW, Kalivas PW, Massie A, Smolders I, Methner A, Pergande M : The cystine/glutamate antiporter system x(c)(−) in health and disease:from molecular mechanisms to novel therapeutic opportunities. Antioxid Redox Signal 2013, 18(5):522-555. 22667998 10.1089/ars.2011.4391 PMC3545354

[11] Bading H: Therapeutic targeting of the pathological triad of extrasynaptic NMDA receptor signaling in neurodegenerations. J Exp Med 2017, 214(3):569–578. 28209726 10.1084/jem.20161673 PMC5339681

[12] Massie A, Boillee S, Hewett S, Knackstedt L, Lewerenz J: Main path and byways: non-vesicular glutamate release by system xc(−) as an important modifier of gluamatergic neurotransmission. J Neurochem 2015, 135(6):1062–1079. 26336934 10.1111/jnc.13348 PMC4762049

[13] Mesci P, Zaidi S, Lobsiger CS, Millecamps S, Escartin C, Seilhean D, Sato H, Mallat M, Boillee S: System xC- is a mediator of microglial function and its deletion slows symptoms in amyotrophic lateral sclerosis mice. Brain 2015, 138(Pt 1):53–68. 25384799 10.1093/brain/awu312 PMC4441079

[14] Mao X, Moerman AM, Lucas MM, Barger SW: Inhibition of the activity of a neuronal kB-binding factor (NKBF) by glutamate. J Neurochem 1999, 73:1851–1858. 10537043

[15] Wu SZ, Bodles AM, Porter MM, Griffi n WS, Basile AS, Barger SW: Induction of serine racemase expression and D-serine release from microglia by amyloid beta-peptide. J Neuroinflammation 2004, 1(1):2. 15285800 10.1186/1742-2094-1-2 PMC483052

[16] Wu SZ, Jiang S, Sims TJ, Barger SW: Schwann cells exhibit excitotoxicity consistent with release of NMDA receptor agonists. J Neurosci Res 2005, 79(5):638–643. 15672444 10.1002/jnr.20401

[17] Brown GC, Vilalta A: How microglia kill neurons. Brain Res 2015, 1628(Pt B):288–297. 26341532 10.1016/j.brainres.2015.08.031

[18] Piani D, Spranger M, Frei K, Schaffner A, Fontana A: Macrophage-induced cytotoxicity of N-methyl-D- aspartate receptor positive neurons involves excitatory amino acids rather than reactive oxygen intermediates and cytokines. Eur J Immunol 1992, 22(9):2429–2436. 1355433 10.1002/eji.1830220936

[19] Qin S, Colin C, Hinners I, Gervais A, Cheret C, Mallat M: System Xc- and apolipoprotein E expressed by microglia have opposite effects on the neurotoxicity of amyloid-beta peptide 1–40. J Neurosci 2006, 26(12):3345–3356. 16554485 10.1523/JNEUROSCI.5186-05.2006 PMC6674113

[20] Bridges RJ, Natale NR, Patel SA: System xc(−) cystine/glutamate antiporter: an update on molecular pharmacology and roles within the CNS. Br J Pharmacol 2012, 165(1):20–34. 21564084 10.1111/j.1476-5381.2011.01480.x PMC3252963

[21] Wu S, Zhou J, Zhang H, Barger SW: Serine Racemase Expression Differentiates Aging from Alzheimer’s Brain. Curr Alzheimer Res 2022, 19(7):494–502. 35929621 10.2174/1567205019666220805105106

[22] Guillemin GJ: Quinolinic acid, the inescapable neurotoxin. FEBS J 2012, 279(8):1356–1365. 22248144 10.1111/j.1742-4658.2012.08485.x

